# Mixed Methods Studies Examining the Physical Activity Practices Among African American and Black Women: Protocol for a Methodological Scoping Review

**DOI:** 10.2196/93012

**Published:** 2026-07-17

**Authors:** Annette Moore Hubbell, Laura Killingsworth, Michele Shropshire

**Affiliations:** 1 Mennonite College of Nursing Illinois State University Normal, IL United States; 2 Milner Library Illinois State University Normal, IL United States

**Keywords:** African American and Black women, physical activity, exercise behavior, mixed methods research, quantitative and qualitative, literature review

## Abstract

**Background:**

African American and Black women are among the least physically active demographic groups in the United States and experience disproportionate burdens of chronic disease that may be reduced through regular physical activity (PA). Mixed methods research is increasingly used to examine the behavioral, social, and contextual factors that influence PA in this population.

**Objective:**

This study aims to identify, examine, and describe the mixed methods designs used in research on PA practices among African American and Black women in the United States; compare methodological approaches; identify gaps in the literature; and provide recommendations for future research.

**Methods:**

This scoping review will follow Joanna Briggs Institute methodology. A 3-step search strategy was developed and peer reviewed using the Peer Review of Electronic Search Strategies guideline. Seven databases will be searched: Academic Search Ultimate, Agricultural and Environmental Science Database, APA PsycINFO, CINAHL Ultimate, PubMed, SocINDEX, and SPORTDiscus. Eligible studies will be primary mixed methods investigations involving non-Hispanic African American or Black women aged ≥18 years residing in the United States. Studies must include both quantitative and qualitative components with evidence of integration. Records will be deduplicated and screened in Rayyan according to population, concept, and context eligibility criteria. Data will be summarized using descriptive statistics, tables, and an evidence map. The review will be limited to English-language studies published from February 1, 2011, through February 18, 2026.

**Results:**

The protocol was registered with the Open Science Framework. The search strategy was piloted, refined, and finalized on February 18, 2026. Database searching has been completed, records have been imported and deduplicated, and screening is currently ongoing. Data extraction and synthesis have not yet commenced. Completion of study selection, data charting, and synthesis is anticipated by August 2026, with submission of the completed scoping review planned thereafter.

**Conclusions:**

This review will provide a methodological map of mixed methods studies examining PA among African American and Black women, including design types, integration strategies, timing, prioritization, use of joint displays, and reporting practices. Findings will inform future mixed methods research and support the development of more rigorous and transparent investigations in this field.

**Trial Registration:**

Open Science Framework 10.17605/OSF.IO/NA9ME; https://osf.io/na9me/overview

**International Registered Report Identifier (IRRID):**

PRR1-10.2196/93012

## Introduction

Regular physical activity (PA) is a cornerstone of chronic disease prevention, yet nearly one-third of adults worldwide fail to meet the recommended guidelines for PA [1]. In the United States, women are less likely than men to meet PA guidelines, and African American and Black women report the lowest activity levels across racial and ethnic groups and across sex and gender categories [2,3]. Persistent inequities in PA contribute to elevated burdens of cardiometabolic disease, even as culturally responsive interventions continue to evolve. Despite the increasing use of mixed methods approaches in health research, there is limited methodological synthesis examining how these approaches are applied specifically to PA research among African American and Black women. Mixed methods approaches may provide essential information on how and why PA patterns persist by integrating quantitative measures of behavior with qualitative insights into beliefs, values, social environments, and structural influences. To guide the primary authors in developing a mixed methods research proposal, it is important to first understand the current state of mixed methods studies examining the PA practices among African American and Black women.

To increase understanding, we define several terms used throughout this protocol. The term “African American” is defined as “an American of African and especially of Black African descent.” [4]. In this protocol, we use “African American and Black” to reflect this definition. Caspersen et al [5] define PA as “any bodily movement produced by skeletal muscles that results in energy expenditure.” This definition includes exercise (a planned form of PA) and physical fitness (a result of PA). Definitions of mixed methods research and mixed methods methodology have evolved over the years. Creswell and Plano Clark [6] examined the emerging features of mixed methods studies, including how researchers used quantitative and qualitative methodologies, and identified the core characteristics of mixed methods research. According to Creswell and Plano Clark [6], “The researcher:

- collects and analyzes both qualitative and quantitative data rigorously in response to research questions and hypotheses,
- integrates (or mixes or combines) the two forms of data and their results,
- organizes these procedures into specific research designs that provide the logic and procedures for conducting the study, and
- frames these procedures within theory and philosophy”

In essence, mixed methods research is defined by the rigorous collection and analysis of both qualitative and quantitative data, the intentional integration of these data, and the organization of these processes within a coherent design and philosophical framework. Core characteristics include explicit mixing procedures, along with transparent justifications for the chosen design and integration strategy [6].

Previous systematic and scoping reviews have examined PA among African American and Black women, primarily focusing on barriers and facilitators [7-9], interventions and their effectiveness [10-12], behavioral determinants [13,14], and health outcomes [15,16]. However, these reviews have not specifically examined the methodological application of mixed methods designs, including how quantitative and qualitative components are integrated. The present scoping review addresses this gap by focusing specifically on the design, integration, and reporting of mixed methods studies in this field.

A scoping review is an appropriate method for mapping the existing literature on this topic. One purpose of a scoping review is to allow researchers to examine the scope and breadth of the literature [17]. For this review, conducting a scoping review of existing studies may help inform the authors about what is known about the topic as they determine the feasibility of developing a mixed methods research proposal.

To our knowledge, no review has systematically mapped the use of mixed methods methodology, including design typologies and integration strategies, in studies of PA among African American and Black women. This scoping review will inform the current state of the science and existing studies with regard to the methods, findings, and limitations. Additionally, we propose that the findings of this scoping review may advance future research and inform recommendations that may distinguish future mixed methods research from previous studies. We will map how mixed methods research has been conceptualized and implemented in studies examining PA practices among African American and Black women to inform future research and promote health equity in this understudied area.

A preliminary search of MEDLINE, the Cochrane Database of Systematic Reviews, and Joanna Briggs Institute (JBI) Evidence Synthesis did not identify any published and/or ongoing scoping or systematic reviews focused on the methodological application of mixed methods in this topic area. Accordingly, this protocol adheres to JBI guidance for scoping reviews and aligns its reporting with the PRISMA-ScR (Preferred Reporting Items for Systematic Reviews and Meta-Analyses Extension for Scoping Reviews) [18] recommendations for transparency in methods (Multimedia Appendix 1).

The aim of this scoping review is to identify, examine, and describe the key characteristics of mixed methods designs in studies examining the PA practices of African American and Black women published from February 1, 2011, through February 18, 2026. This review protocol emphasizes an evidence-based approach and purposefully uses a structured, technology-assisted approach. This review will not critically appraise study quality; rather, it will document and describe reported procedures to map current methodological practice and guide future research design.

## Methods

Reporting will follow the PRISMA-ScR checklist [18], which will be completed and submitted as a multimedia appendix.

### Design and Guidance

This scoping review protocol adheres to the JBI Manual for Evidence Synthesis for scoping reviews [17] and will be reported in accordance with the PRISMA-ScR guidance adapted for scoping review protocols. The protocol is registered with the Open Science Framework (OSF). A search in PROSPERO indicated that scoping review protocols of this type are typically not registered there, and no relevant registrations were identified.

### Eligibility Criteria (Population, Concept, and Context Framework)

The population, concept, and context (PCC) framework is recommended by the JBI for developing scoping review questions [19]. This framework guides the following research question: what is known about the application of mixed methods approaches in research examining the PA practices of African American and Black women residing in the United States?

#### Population

Adults aged ≥18 years who identify as non-Hispanic African American or Black women residing in the United States will be included. For this review, “social classification” refers to the categorization of participants into socially constructed population groups, including race or ethnicity and sex or gender, consistent with National Institutes of Health (NIH) guidance that race and ethnicity are social constructs used to define population groups in health research [20]. Studies will be considered eligible if they report participants’ social classification when outcomes are disaggregated or stratified such that findings specific to African American and Black women can be independently extracted. Studies with mixed samples will be eligible if (1) ≥50% of participants identify as African American or Black women or (2) results are stratified to allow extraction of outcomes for this subgroup. Studies in which African American and Black women are included but results are not disaggregated will be excluded. The ≥50% threshold was selected to ensure that study design and integration strategies meaningfully reflect African American and Black women as the primary population of interest while allowing the inclusion of studies that include limited comparison groups. Both healthy populations and populations with chronic conditions will be included if PA practices are the primary outcome. Exclusion criteria include men; participants aged <18 years, studies that do not report results for African American and Black women, competitive athlete or sport-specific samples that are not reflective of general PA practices, and samples explicitly limited to individuals medically deemed unable to engage in PA (eg, advanced physical frailty or severe neurocognitive disorder).

#### Concept

Primary mixed methods studies will be classified using the Creswell and Plano Clark [6] and Creswell et al [21] typology (eg, convergent, explanatory sequential, exploratory sequential, or other or unspecified) based on explicit design labeling or on designs inferred from study procedures (including the integration of quantitative and qualitative data, timing, priority, and integration points) used to examine PA practices (eg, exercise, physical fitness, or general PA behavior). A study will be considered a “mixed methods” study if it includes both qualitative and quantitative data collection and analysis and demonstrates explicit integration (eg, merging, connecting, or joint interpretation) [6,21].

#### Context

We focus on the United States because of the sociocultural, environmental, and health care policy contexts that shape methodological choices and integration strategies that influence PA engagement among African American and Black women and because this focus supports actionable implications for US-based practice and research. This focus is noted as a limitation; future work may extend this research internationally.

### Study Types

Peer-reviewed, single mixed methods studies (including randomized, nonrandomized, or observational quantitative components paired with rigorous qualitative components, such as phenomenology, grounded theory, ethnography, qualitative description, action research, and feminist research) will be included. This approach is intended to reliably capture evidence of design, integration, and mixing. The exclusion of commentaries, dissertations, conference abstracts, and other gray literature is intended to ensure methodological transparency, peer-reviewed rigor, and the feasibility of the review process. We acknowledge that these exclusions (eg, gray literature) may omit emerging or innovative methodological work. Hence, authors will annotate this limitation in the published scoping review manuscript.

### Time Frame and Language

Studies published from February 1, 2011, through February 18, 2026 (15 years), will be included to capture the rapid growth and increasing methodological sophistication of mixed methods research during this period while maintaining feasibility. Only papers published in the English language will be considered because of reviewer proficiency.

### Information Sources

We will search the following databases: Academic Search Ultimate (EBSCOhost), Agricultural and Environmental Science Database (AESD; ProQuest), APA PsycInfo (ProQuest), CINAHL Ultimate (EBSCOhost), PubMed, SocINDEX (EBSCOhost), and SPORTDiscus (EBSCOhost). Reference lists of included studies will also be screened to identify additional studies. AESD was included to capture community and environmental determinants of PA (eg, built and green space), for which relevant mixed methods studies may be indexed outside traditional health databases.

### Search Strategy

Guided by the JBI Manual’s 3-step process, we conducted a preliminary search in CINAHL and PubMed to refine keywords and subject headings, developed database-specific strategies, and will hand-search the reference lists of included studies. Search terms were informed by previous mixed methods review literature [22,23]. Full search strategies for all databases were developed by one of the authors (a health sciences librarian) using the Peer Review of Electronic Search Strategies (PRESS) guideline and are presented in Multimedia Appendix 2 [24]. All searches will be limited to English and the specified date range. Search results will be uploaded to Zotero (version 7.0.24), a citation management system [25], where duplicates will be removed. Search outputs and deduplication counts will be documented. Potentially relevant sources will be retrieved in full, and their citation details will be imported into Rayyan (Rayyan Systems Inc) systematic review software [26]. Duplicates will also be checked within Rayyan as a secondary step.

### Selection Process

We will conduct a pilot screening of 15 random records to calibrate inclusion and exclusion decisions using a standardized eligibility and definitions guide (Multimedia Appendix 3). Two reviewers will independently screen titles and abstracts as well as full-text articles. Two reviewers will conduct data extraction using a collaborative approach, with discrepancies resolved through discussion or consultation with a third reviewer when needed (AMH).

Multimedia Appendix 4 presents the current PRISMA-ScR flow diagram [27,28], documenting search retrieval and deduplication. Screening and eligibility assessment remain ongoing.

### Data Extraction

Data extraction will be conducted using a modified JBI data extraction instrument [17,29-31] (Multimedia Appendix 5). The data extraction table is structured to comprehensively capture the essential elements of each study included in the scoping review. It begins with scoping review details, including the review title, objectives, and guiding questions, thereby establishing the framework for the review’s focus and scope. Two reviewers will independently pilot the extraction form on an initial sample of studies and compare results to achieve at least 75% agreement. Following calibration, data extraction will proceed collaboratively, with discrepancies resolved through discussion or consultation with a third senior reviewer (AMH). Any modifications to the extraction form will be documented with the date, rationale, and description of the changes and reported in the final manuscript. Study corresponding authors will be contacted for clarification and/or to obtain missing information when necessary and/or feasible.

### Data Items

We will capture citation details, setting, PA focus, study objectives, rationale for using mixed methods, participant demographics and sample characteristics, mixed methods design (convergent, explanatory sequential, exploratory sequential, or not specified) [6], quantitative and qualitative data collection and analysis methods, reliability and trustworthiness indicators, integration methods (merging, connecting, or embedding) and evidence of mixing (transformation, comparison, and synthesis) [6,32,33], the presence and type of visual integration tools (eg, joint displays) [23], key findings and meta-inferences related to our review question, strengths and limitations, funding, and authors’ recommendations. Integration will be evaluated descriptively by identifying (1) the type of integration (merging, connecting, or embedding), (2) the stage of integration (design, methods, or interpretation), and (3) the presence of integration products (eg, joint displays and meta-inferences). We will not score quality but will document the extent and transparency of integration reporting.

### Synthesis and Presentation of Results

We will produce (1) a narrative synthesis summarizing patterns in design choice, integration strategies, and evidence of mixing; (2) a tabular summary mapping design types to methods and integration techniques (Multimedia Appendix 6); and (3) a color-coded frequency flow visualizing design prevalence and evidence of mixing (Multimedia Appendix 7). No critical appraisal will be undertaken, in line with JBI guidance for scoping reviews [17].

### Patient and Public Involvement

No patients or members of the public will be involved in conducting this methodological scoping review.

## Results

This is a protocol; therefore, no study findings are reported. The preliminary search strategy was piloted on September 11, 2025, and subsequently refined. The final pilot search was conducted on February 18, 2026. We anticipate finalizing search updates in March 2026. Screening and study selection were completed by April 2026. Completion of data charting and synthesis is anticipated by August 2026 with submission of the completed scoping review manuscript thereafter.

## Discussion

### Principal Findings

This scoping review is expected to provide the first comprehensive methodological map of mixed methods studies examining PA among African American and Black women published from February 1, 2011, through February 18, 2026. We anticipate identifying substantial variability in mixed methods design selection, integration procedures [33], reporting transparency, and the use of integration products such as joint displays and meta-inferences [23,32]. This review will clarify how researchers have combined quantitative and qualitative approaches to understand PA practices and related contextual influences in this population.

By focusing on methodological characteristics, such as integration procedures, the use of joint displays, and the reporting of meta‑inferences, the review will describe current methodological practices and identify opportunities for further development in future research. It will also identify areas in which mixed methods designs can be more fully implemented, such as by clarifying how mixing occurs or by more clearly describing the rationale for design selection.

### Comparison With Prior Work

Previous reviews examining PA among African American and Black women have primarily focused on intervention effectiveness, barriers and facilitators, behavioral determinants, and health outcomes. However, these reviews have not evaluated how mixed methods approaches were designed, conducted, and integrated. By focusing on methodological characteristics rather than substantive intervention outcomes, the present review addresses an important gap in the literature and extends understanding of methodological practices in this field.

### Strengths and Limitations

Strengths include adherence to JBI methodology; a transparent, preregistered protocol; librarian-supported database searching; independent screening procedures; and explicit attention to mixed methods integration practices. Limitations include the restriction to English-language publications, US-based studies, and peer-reviewed literature, which may exclude relevant international or unpublished methodological work. Furthermore, consistent with scoping review methodology, no formal critical appraisal or risk-of-bias assessment will be conducted.

### Future Directions

Findings from this review may inform future mixed methods investigations by identifying opportunities to improve (1) the clear reporting of mixed methods frameworks; (2) the clear reporting of integration and the use of joint displays; and (3) equity-informed designs that better capture the social, cultural, and structural factors influencing PA among African American and Black women.

### Dissemination Plan

Findings will be disseminated through peer-reviewed publications, conference presentations, and integration into future grant development and educational materials.

### Conclusions

This protocol describes a systematic approach to mapping the use of mixed methods research in studies of PA among African American and Black women. The completed review is expected to provide a methodological overview of design and integration practices, identify gaps in reporting, and inform future mixed methods research in this area. These findings may contribute to the development of more rigorous and transparent research focused on promoting PA and health equity.

## Appendix

Multimedia Appendix 1 PRISMA-ScR checklist.

Multimedia Appendix 2 Search strategy.

Multimedia Appendix 3 Study selection process.

Multimedia Appendix 4 PRISMA (Preferred Reporting Items for Systematic Reviews and Meta-Analyses) flow diagram.

Multimedia Appendix 5 Data extraction instrument.

Multimedia Appendix 6 Data analysis table.

Multimedia Appendix 7 Frequency flow diagram.

## Abbreviations

AESD: Agricultural and Environmental Science Database
JBI: Joanna Briggs Institute
NIH: National Institutes of Health
OSF: Open Science Framework
PA: physical activity
PCC: population, concept, and context
PRESS: Peer Review of Electronic Search Strategies
PRISMA-ScR: Preferred Reporting Items for Systematic Reviews and Meta-Analyses Extension for Scoping Reviews

## Data Availability

All materials associated with this protocol (eg, search strategies and data extraction templates) are available on the Open Science Framework and in the multimedia appendices accompanying this manuscript.
