# Supplementary material for: Mixed Methods Studies Examining the Physical Activity Practices Among African American and Black Women: Protocol for a Methodological Scoping Review
Source: JMIR Res Protoc. 2026 Jul 17;15:e93012. doi: 10.2196/93012 (PMC13428207; doi:10.2196/93012)
Supplement: Multimedia Appendix 2 [file resprot_v15i1e93012_app2.docx]

Appendix II

Search Strategy

**Academic Search Ultimate**

S1: ((XB (black) OR XB "african american" OR DE "African Americans" OR DE "Black people") AND (DE "Women" OR XB (women))) OR (DE "African American women" OR DE "Black women")

S2: DE "Exercise" OR DE "Physical activity" OR DE "Aerobic exercises" OR DE "Physical fitness" OR XB (exercis*) OR XB "physical activity" OR XB (fitness) OR XB "aerobic exercis*" OR XB "aerobic training"

S3: TX "mixed method*" OR TX "mixed-method*" OR TX "convergent parallel" OR TX "convergent design" OR TX "explanatory sequential" OR TX "explanatory design" OR TX "exploratory sequential" OR TX "exploratory design" OR TX "embedded design" OR TX (multimethod) OR (TX (qualitative AND quantitative))

English, Scholarly Journals, 2011/02/01 to 2026/02/18

**Agricultural & Environmental Science Database**

S1: noft(black AND woman) OR noft("african american" AND women) OR noft("black women") OR noft("african american women")

S2: noft(exercis* OR "physical activity" OR fitness OR "aerobic exercis*" OR "aerobic training")

S3: "mixed method*" OR "mixed-method*" OR "convergent parallel" OR "convergent design" OR "explanatory sequential" OR "explanatory design" OR "exploratory sequential" OR "exploratory design" OR "embedded design" OR (multimethod) OR (qualitative AND quantitative)

English, Scholarly Journals, 2011/02/01 to 2026/02/18

**APA PsycINFO**

S1: (MAINSUBJECT.EXACT("Black People") AND MAINSUBJECT.EXACT("Human Females")) OR noft((black AND women) OR ("african american" AND women) OR "black women" OR "african american women")

S2: MAINSUBJECT.EXACT("Exercise") OR MAINSUBJECT.EXACT("Physical Activity") OR MAINSUBJECT.EXACT("Aerobic Exercise") OR MAINSUBJECT.EXACT("Physical Fitness") OR noft(exercis*) OR noft("physical activity") OR noft("aerobic exercis*") OR noft(fitness) OR noft("aerobic training")

S3: "mixed method*" OR "mixed-method*" OR "convergent parallel" OR "convergent design" OR "explanatory sequential" OR "explanatory design" OR "exploratory sequential" OR "exploratory design" OR "embedded design" OR (multimethod) OR (qualitative AND quantitative)

English, Scholarly Journals, 2011/02/01 to 2026/02/18

**CINAHL**

S1: ((MH "African Americans" OR MH "Black Persons" OR XB "african american" OR XB "black") AND (MH "Women" OR XB "women"))

S2: MH "Exercise" OR MH "Physical Activity" OR MH "Aerobic Exercises" OR MH "Physical Fitness" OR XB "physical activity" OR XB "fitness" OR XB "exercis*" OR XB "aerobic exercis*" OR XB "aerobic training"

S3: TX "mixed method*" OR TX "mixed-method*" OR TX "convergent parallel" OR TX "convergent design" OR TX "explanatory sequential" OR TX "explanatory design" OR TX "exploratory sequential" OR TX "exploratory design" OR TX "embedded design" OR TX (multimethod) OR (TX (qualitative AND quantitative))

English, Scholarly Journals, 2011/02/01 to 2026/02/18

**PubMed**

S1: ((((black[Title/Abstract] AND women[Title/Abstract]) OR ("african american"[Title/Abstract] AND women[Title/Abstract])) OR ("black women"[Title/Abstract])) OR ("african american women"[Title/Abstract])) OR ("Black or African American"[Mesh] AND Women[Mesh])

AND

S2: (((((Exercise[Mesh]) OR ("physical activity"[Title/Abstract])) OR ("physical exercise"[Title/Abstract])) OR (exercis*[Title/Abstract])) OR (fitness[Title/Abstract])) OR ("aerobic exercise"[Title/Abstract])

AND

S3: (((((((((("mixed method*"[Title/Abstract]) OR ("mixed-method*"[Title/Abstract])) OR ("convergent parallel"[Title/Abstract])) OR ("convergent design*"[Title/Abstract])) OR ("explanatory sequential"[Title/Abstract])) OR ("explanatory design"[Title/Abstract])) OR ("exploratory design"[Title/Abstract])) OR ("exploratory sequential"[Title/Abstract])) OR ("embedded design"[Title/Abstract])) OR (multimethod[Title/Abstract])) OR ((qualitative[Title/Abstract] AND quantitative[Title/Abstract]))

English, February 1, 2011, through February 18, 2026.

**SocINDEX**

S1: ((XB (black) OR XB "african american" OR DE "AFRICAN Americans" OR DE "BLACK people") AND (DE "WOMEN" OR XB (women))) OR (DE "AFRICAN American women" OR DE "BLACK women")

S2: DE "EXERCISE" OR DE "EXERCISE & psychology" OR DE "PHYSICAL fitness" OR XB (exercis*) OR XB "physical activity" OR XB (fitness) OR XB "aerobic exercis*" OR XB "aerobic training"

S3: TX "mixed method*" OR TX "mixed-method*" OR TX "convergent parallel" OR TX "convergent design" OR TX "explanatory sequential" OR TX "explanatory design" OR TX "exploratory sequential" OR TX "exploratory design" OR TX "embedded design" OR TX (multimethod) OR (TX (qualitative AND quantitative))

English, Academic Journals, 02/2011 to 02/2026

**SPORTDiscus**

S1: XB ((black AND women) OR ("african american" AND women) OR "black women" OR "african american women")

S2: DE "EXERCISE" OR DE "PHYSICAL activity" OR DE "AEROBIC exercises" OR DE "PHYSICAL fitness" OR DE "EXERCISE physiology" OR DE "EXERCISE & psychology" OR DE "EXERCISE physiology" OR XB (exercis*) OR XB "physical activity" OR XB (fitness) OR XB "aerobic exercis*" OR XB "aerobic training"

S3: TX "mixed method*" OR TX "mixed-method*" OR TX "convergent parallel" OR TX "convergent design" OR TX "explanatory sequential" OR TX "explanatory design" OR TX "exploratory sequential" OR TX "exploratory design" OR TX "embedded design" OR TX (multimethod) OR (TX (qualitative AND quantitative))

English, Academic Journals, 02/2011 to 02/2026
