# Supplementary material for: Mixed Methods Studies Examining the Physical Activity Practices Among African American and Black Women: Protocol for a Methodological Scoping Review
Source: JMIR Res Protoc. 2026 Jul 17;15:e93012. doi: 10.2196/93012 (PMC13428207; doi:10.2196/93012)
Supplement: Multimedia Appendix 3 [file resprot_v15i1e93012_app3.docx]

Appendix III

Table 1. Exemplar of the study selection process for titles and abstracts

| **Eligibility Criteria** | | | | | **List reason(s) for exclusion** |
| --- | --- | --- | --- | --- | --- |
| Author | Sample:  -Non-Hispanic AA/Black women living in the United States  -Aged > 18  -> 50% of participants are AA/Black women  and results reported by classification  -Participants able to partake in PA | Examining a form of PA, e.g., exercise, physical fitness  (yes/no) | Study identified as Mixed Methods | |  |
|  |  |  | Contains a quantitative method  (yes/ no) | Contains a qualitative method  (yes/ no) |  |

*Note.* AA: African American
