# Supplementary material for: Mixed Methods Studies Examining the Physical Activity Practices Among African American and Black Women: Protocol for a Methodological Scoping Review
Source: JMIR Res Protoc. 2026 Jul 17;15:e93012. doi: 10.2196/93012 (PMC13428207; doi:10.2196/93012)
Supplement: Multimedia Appendix 4 [file resprot_v15i1e93012_app4.docx]

Appendix IV

Prisma Flow Diagram

**Identification of studies via databases**

Records identified from:

Academic Search Ultimate (n = 125)

AES: (n = 94)

APA PsycINFO (n = 33)

CINAHL (n = 133)

PubMed (n = 87)

SocINDEX(n = 12)

SPORTDiscus (n = 4)

Preliminary search (n= 50)

Records removed *before screening*:

Duplicate records removed (n = 327)

**Identification**

Records screened

(n = 211)

Records excluded

(n = 163)

Reports not sought for retrieval

(n = 16)

Reports sought for retrieval

(n = 48)

**Screening**

Reports assessed for eligibility

(n = 32)

Reports excluded:

Wrong population (n= 4)

Wrong study design (n = 5)

Conference abstract (n = 3)

Wrong indication (n= 3)

Wrong outcome (n = 1)

etc.

Studies included in review

(n = 16)

**Included**

**Figure 1** PRISMA flow diagram summarizing the process for identifying and screening reviews for inclusion in the scoping review. (Tricco et al., 2018; Page et al., 2021)

Source: Page MJ, et al. BMJ 2021;372:n71. doi: 10.1136/bmj.n71.
