# Supplementary material for: Mixed Methods Studies Examining the Physical Activity Practices Among African American and Black Women: Protocol for a Methodological Scoping Review
Source: JMIR Res Protoc. 2026 Jul 17;15:e93012. doi: 10.2196/93012 (PMC13428207; doi:10.2196/93012)
Supplement: Multimedia Appendix 5 [file resprot_v15i1e93012_app5.docx]

Appendix V

Table 2. Data extraction instrument

| **Scoping Review Details** | | | | | | |
| --- | --- | --- | --- | --- | --- | --- |
| Scoping Review Title  Review Objectives  Review Questions | | | | | | |
| **Inclusion/Exclusion** **Criteria** | | | | | | |
| Population  Concept  Context  Types of evidence source | | | | | | |
| **Evidence Source Details and Characteristics** | | | | | | |
| Citation details (e.g. author/s, date, title, journal, volume, issue, pages) | Location and/or setting | PA Focus | Study Objectives | Rationale for MM | Participant  Demographics  Sample #  Age  Identify as AA/Black (%) | Intervention type, if applicable |
|  |  |  |  |  |  |  |
|  |  |  |  |  |  |  |

| **Evidence Source Details and Characteristics (continued)** | | | | | | |
| --- | --- | --- | --- | --- | --- | --- |
| Mixed Methods Design: (convergent, explanatory sequential, exploratory sequential, not specified) | | | | | | |
| Quantitative Method: | | | Qualitative Method: | | | Integration  Methods: (merging, connecting, embedding, unspecified)  Or Evidence of mixing the data: (transformation, comparison, synthesis, unspecified) |
| Data Collection | Data Analysis | Reliability | Data Collection | Data Analysis | Trustworthiness |  |
|  |  |  |  |  |  |  |
|  |  |  |  |  |  |  |

| **Evidence Source Details and Characteristics (continued)** | | | | |
| --- | --- | --- | --- | --- |
| Visual Tools Used to Illustrate Integration (e.g., joint display)?  (yes/no) | Key findings/meta inferences related to the review question | Strengths/  Limitations | Funding Source | Recommendations |
|  |  |  |  |  |
|  |  |  |  |  |

*Note*. PA= physical activity; AA= African American; MM= mixed methods
