# Supplementary material for: Mixed Methods Studies Examining the Physical Activity Practices Among African American and Black Women: Protocol for a Methodological Scoping Review
Source: JMIR Res Protoc. 2026 Jul 17;15:e93012. doi: 10.2196/93012 (PMC13428207; doi:10.2196/93012)
Supplement: Multimedia Appendix 6 [file resprot_v15i1e93012_app6.docx]

Appendix VI

Table 3. Data analysis table

| **Review question:** What is known about the application of mixed methods approaches in research examining the PA practices of AA women? | | | | | |
| --- | --- | --- | --- | --- | --- |
| Citation details: Author/s & year | Rationale for MM | Type of Mixed Methods Design | Integration Method | Evidence of Mixing | Strengths & Limitations |
|  |  |  |  |  |  |
|  |  |  |  |  |  |

*Note.* MM= mixed methods
