# Supplementary material for: Mixed Methods Studies Examining the Physical Activity Practices Among African American and Black Women: Protocol for a Methodological Scoping Review
Source: JMIR Res Protoc. 2026 Jul 17;15:e93012. doi: 10.2196/93012 (PMC13428207; doi:10.2196/93012)
Supplement: Multimedia Appendix 7 [file resprot_v15i1e93012_app7.docx]

Appendix VII

Table 4. Frequency flow diagram of mixed methods designs


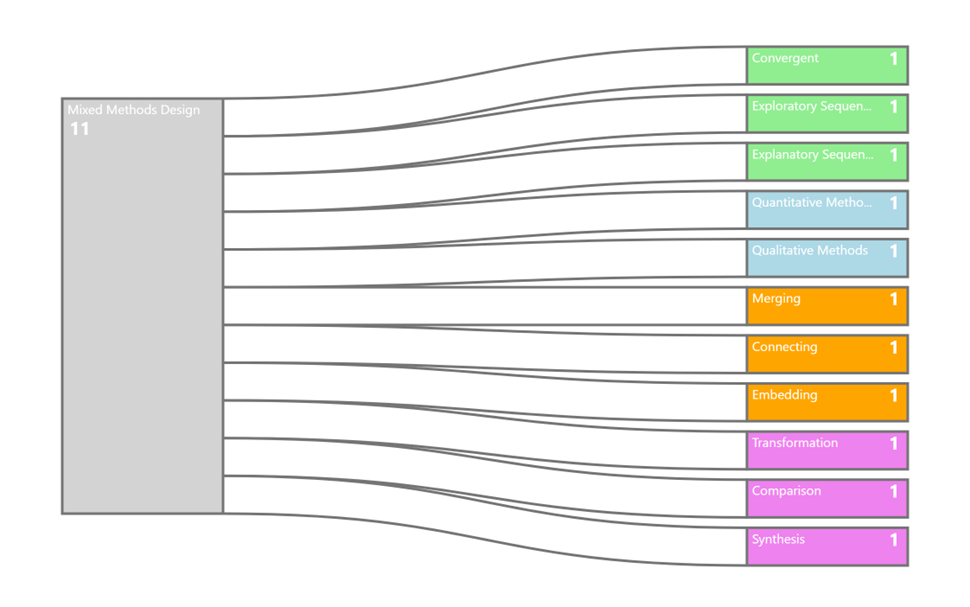


*Note.* Grey = mixed method designs included in the review. Green = design method.

Blue = quantitative and qualitative methods. Orange = integration method. Violet = evidence of mixing. The number denotes the number of studies in each branch.
